# Receiving results of uncertain clinical relevance from population genetic screening: systematic review & meta-synthesis of qualitative research

**DOI:** 10.1038/s41431-022-01054-5

**Published:** 2022-03-08

**Authors:** Faye Johnson, Fiona Ulph, Rhona MacLeod, Kevin W. Southern

**Affiliations:** 1 Division of Psychology and Mental Health, School of Health Sciences, University of Manchester, Manchester, UK; 2 Division of Evolution, Infection and Genomics, School of Biological Sciences, The University of Manchester, Manchester, UK; 3 Manchester Centre for Genomic Medicine, St Mary’s Hospital, Manchester University Hospitals NHS Foundation Trust, Manchester, UK; 4 Institute of Life Course and Medical Sciences, University of Liverpool, Liverpool, UK

**Keywords:** Diagnosis, Psychology, Health policy, Ethics, Genetic counselling

## Abstract

Genetic screening can be hugely beneficial, yet its expansion poses clinical and ethical challenges due to results of uncertain clinical relevance (such as ‘cystic fibrosis screen positive, inconclusive diagnosis’/CFSPID). This review systematically identifies, appraises, and synthesises the qualitative research on experiences of receiving results of uncertain clinical relevance from population genetic screening. Eight databases were systematically searched for original qualitative research using the SPIDER framework, and checked against inclusion criteria by the research team and an independent researcher. Nine papers were included (from USA, Canada, UK, New Zealand). PRISMA, ENTREQ, and EMERGE guidance were used to report. Quality was appraised using criteria for qualitative research. All papers focused on parental responses to uncertain results from newborn screening. Data were synthesised using meta-ethnography and first- and second-order constructs. Findings suggest that results of uncertain clinical relevance are often experienced in the same way as a ‘full-blown’ diagnosis. This has significant emotional and behavioural impact, for example adoption of lifestyle-altering disease-focused behaviours. Analysis suggests this may be due to the results not fitting a common medical model, leading recipients to interpret the significance of the result maladaptively. Findings suggest scope for professionals to negotiate and reframe uncertain screening results. Clearer initial communication is needed to reassure recipients there is no immediate severe health risk from these types of results. Public understanding of an appropriate medical model, that accounts for uncertain genetic screening results in a non-threatening way, may be key to maximising the benefits of genomic medicine and minimising potential psychological harm.

## Introduction

Screening is a public health initiative for early identification, diagnosis and treatment of conditions [1]. According to World Health Organisation criteria, conditions must be clinically actionable to warrant early diagnosis, and the overall benefits of screening should outweigh the cost [1, 2]. Screening can be whole population (for example, newborn screening for phenylketonuria) or sub-populations of increased risk (for example, hereditary cancers) [3]. Developments in screening are bringing in ‘the genomic era’ [4], heralding expanded NBS and next-generation sequencing (NGS) [4]. This has the potential to improve outcomes in many cases [4]. However, this would also increase identification of uncertain results, including incidental findings [5, 6] and variants of uncertain significance [7]. Screening may also uncover results with limited understanding of clinical implications. There may be ethical challenges regarding uncertain diagnoses and prognoses, and the impact of making individuals aware of diseases which may never manifest [8]. These include the social harms of conferring ‘the sick role’ [9], distress and anxiety, unnecessary medical tests/interventions, and increased pressure on healthcare services [8, 9]. These issues are key to debates about expanded population screening and introducing NGS [6]. Do benefits of extra diagnostic power outweigh the cost of identifying results of uncertain clinical relevance in more people? We sought to look at uncertain results across programmes, however, the studies that met eligibility criteria related specifically to newborn screening. This paper will therefore focus on results of uncertain clinical relevance from newborn bloodspot screening (NBS).

### Population genetic screening

A key element of population screening is that there may not be undue reason to suspect or prepare for an abnormal result [10]. This differs from uncertain results that can be returned from diagnostic genetic testing (e.g. for hereditary cancers) [11], as testing is usually sought due to family history or symptoms, and the individual is prepared for possible outcomes [3, 12]. Thus, these experiences have limited transferability to genomic screening. We therefore focus on population genetic screening of individuals without prior awareness of risk, as the psychological impact and ethical responsibilities differ [3, 10]. We chose not to search solely for NBS/adult programmes, as there is presently little evidence in a screening context so a broad approach was felt appropriate. However, all eligible papers were NBS studies, suggesting there may be features unique to NBS [13, 14].

### Qualitative approach

To our knowledge, there are currently no published systematic reviews of the qualitative literature on this topic. Whilst quantitative research allows outcomes to be quantifiable and applicable to policy, it risks excluding important evidence which is not so easily summarised [15]. Qualitative methods have a special potential for in-depth interrogation of unique perspectives [15], which can aid understanding of impact and drive policy change.

### Aims

This review aims to systematically search and appraise qualitative research, and synthesise experiences of receiving results of uncertain clinical relevance from NBS. We aim to propose an explanatory theory of what may underpin these experiences, which could help inform debates regarding the merits and concerns of genomic screening. We aim to identify directions for future research and practice.

## Methods

This review is registered on PROSPERO (CRD42020197750) & reported according to ENTREQ [16] & EMERGE [17] guidance.

### Search strategy and eligibility criteria

**Fig. 1:**
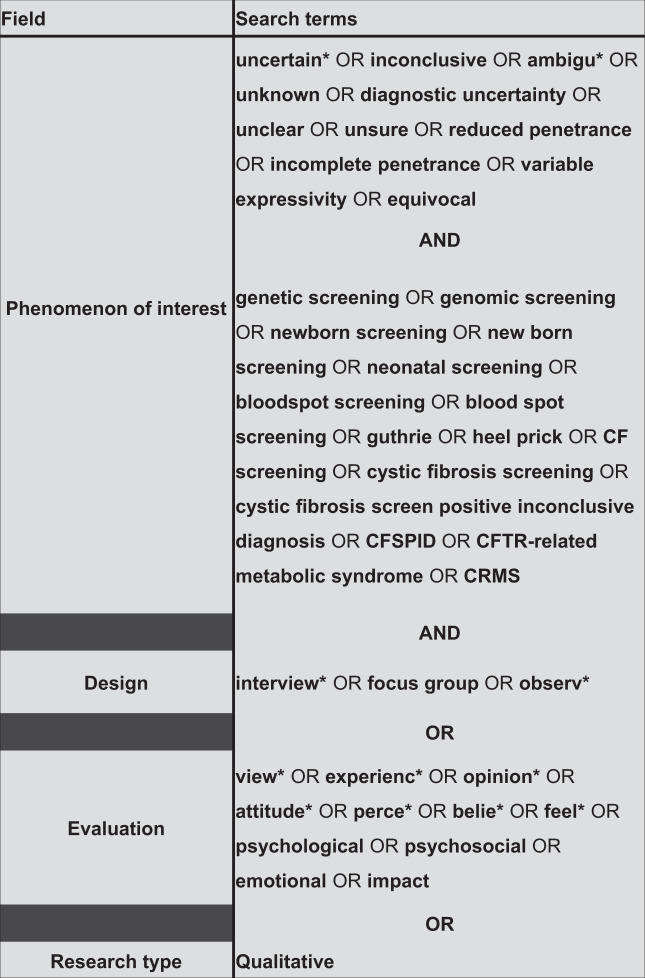
Search. The left panel shows the dimensions of the SPIDER [18] that were used to develop the search. Search terms and Boolean operators are shown in the right. How these were entered was adapted if necessary for the conventions of the different databases.

**Fig. 2:**
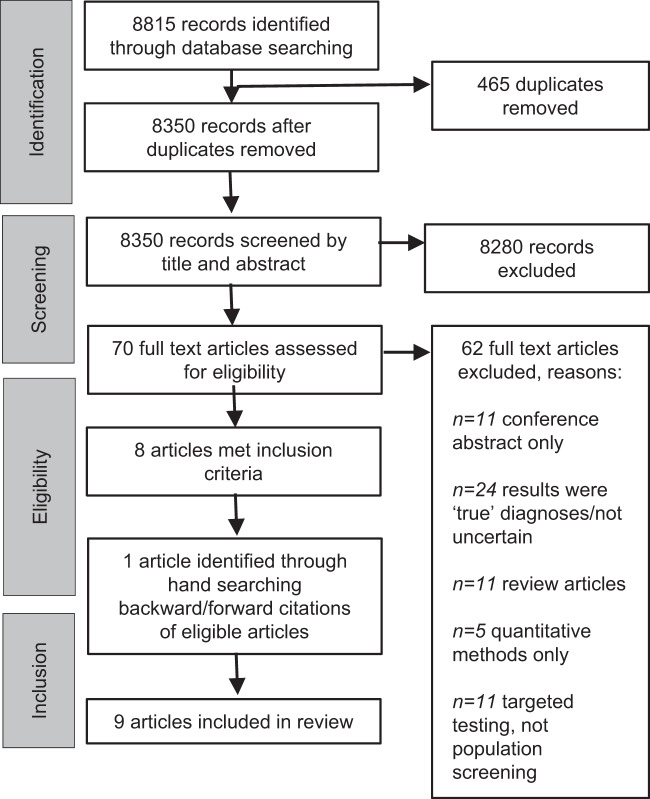
PRISMA diagram. Flow diagram illustrating how articles were identified and selected, with reasons.

Electronic databases were searched up to the 6th of June 2020: MEDLINE (Ovid), PsycINFO (Ovid), EMBASE (Ovid), CINAHL (EBSCO), Web of Science (Clarivate), Dissertations and Theses Global (ProQuest), NHS Evidence, and OpenGrey. The year of publication was not restricted. Search terms were developed using the SPIDER tool [18]. Terms related to uncertainty, genetic screening, and potential sources of uncertainty in genetics were used (Fig. 1). Articles were judged against eligibility criteria (Table 1) and hand searched for citations (Fig. 2). The first author screened titles, abstracts, and full texts of all potential articles. An independent researcher screened texts for validity (75% agreement). The process was documented and discussed until all authors agreed.

**Table 1:** Eligibility criteria for papers.

Inclusion criteria	Exclusion criteria
• Original research articles.	• Literature reviews, meta-analyses or meta-syntheses.
• Qualitative methods of data collection and analysis.	• Quantitative methods only.
• Studies of the impact of uncertain results from population genetic screening.	• Studies of the impact of results from predictive genetic testing.
• Any recipients of uncertain screening results (including parents of screened children).	• Studies of the impact of results from targeted genetic testing for individuals.
• Any country.	
• Published in English.

### Quality appraisal

Included papers were critically appraised using a checklist based in subjectivist epistemology for use with qualitative literature [19]. As a reflective tool rather than a prescriptive rating system [19], the papers’ quality did not determine their inclusion in the review although it did inform interpretation. Flaws may be considered significant if they affect studies’ credibility, transferability, dependability, and confirmability [20]. Dimensions considered are: ‘Scope and purpose’; ‘Design’; ‘Analysis’; ‘Interpretation’; ‘Reflexivity’; ‘Ethical dimensions’ and ‘Relevance and transferability’ [19].

### Data extraction

Main characteristics were extracted (Table 2). Data for synthesis were study authors’ key metaphors, phrases, ideas, and concepts (Table 3).

**Table 2:** Main characteristics of included papers.

Authors (year)	Data collection	Data analysis	Country	Participants	Screening result
Timmermans and Buchbinder (2010) [24]	Observations & open-ended interviews.	Modified grounded theory & analytical-induction tradition.	USA	Families (*n* = 55) of infants(total). 24 infants had uncertain results.	“Deeply ambiguous” newborn screening (NBS) results for various metabolic disorders [e.g. 3-methylcrotonyl-CoA carboxylase deficiency (3-MCC); medium chain acyl-coA dehydrogenase deficiency (MCADD)].
Timmermans and Buchbinder (2012) [25]	Observations.	Modified grounded theory & analytical-induction tradition.	USA	Families of clinic patients (*n* = 75)	Medium chain acyl-coA dehydrogenase deficiency (MCADD).
DeLuca et al. (2011) [26]	Semi-structured open-ended interviews.	Qualitative content analysis.	USA	Parents (*n* = 44) (30 families) (total). 13 infants had uncertain results.	Equivocal NBS results included: persistently abnormal biochemical metabolites (eg. prolonged elevated tyrosine), molecular variants of uncertain significance (eg. isovaleric acidemia ‘NBS variant’), & enzyme deficiencies of uncertain clinical risk (eg. galactocerebrosidase deficiency [Krabbe disease]).
Tluczek et al. (2010) [27]	Open-ended interviews (secondary analysis of interviews from larger mixed-methods study).	Grounded dimensional analysis.	USA	Parents (*n* = 10) of 5 infants.	Equivocal results for cystic fibrosis (CF) (abnormal NBS results plus two CF gene mutations and a normal or intermediate sweat test).
Hayeems et al. (2017) [28]	Mixed-methods (questionnaire & semi-structured open-ended interviews).	Thematic analysis of interviews.	Canada	Parents (*n* = 20) of 18 infants (16 mothers, 2 couples).	Inconclusive results for cystic fibrosis (CF).
Sadat et al. (2020) [29]	Mixed-methods (biomedical records; health status survey; Paediatric Inventory for Parents (PIP); interviews).	No qualitative analysis method for the interviews explictly specified.	USA	Parents of children (*n* = 60) (total). For interviews, parents of children (*n* = 18).	Short-chain acyl-CoA dehydrogenase deficiency (SCADD) or isobutyryl-CoA dehydrogenase deficiency (IBDD)
Johnson et al. (2019) [31]	Semi-structured interviews.	Interpretative phenomenological analysis (IPA)	UK	Parents (*n* = 8) (5 families) (2 mothers, 3 couples).	Cystic fibrosis screen positive, inconclusive diagnosis (CFSPID).
Kerruish (2011) [32]	Semi-structured interviews.	Interpretative phenomenological analysis (IPA)	New Zealand	Parents (*n* = 11) (9 mothers, 1 couple).	Increased genetic risk of type 1 diabetes (T1D) (“one in 16 risk compared to the general population risk of one in 300”).
Tluczek et al. (2019) [30]	Mixed-methods (biomedical records; self-report scales; 10–15 min interviews consisting of 3 open-ended questions).	Content analysis of interviews.	USA	Parents (*n* = 110) (total). With uncertain results: parents (*n* = 20) of 16 children.	Intermediate cystic fibrosis (CF) classification (abnormal NBS results for CF & sweat chloride levels between 30 and 59 mmol/L).

**Table 3:** Content of included papers pertaining to the key concepts from the analysis.

Authors (year)	Uncertainty and identity	Emotional response	Behavioural response	Cognitive response	Medicine and the role of professionals	Individual differences, communication and information needs
Timmermans and Buchbinder (2010) [24]	“Patients in waiting”; “illness identity”; “A liminal state between pathology and normalcy”; “Living between health and disease”; “not sick, but not normal”	Shock; “a roller coaster ride”	“Precautions”; “actions that prove the social relevancy of [the condition]”; “preventative practices” as “measures to offset the ‘real’ disease”		“Shared patient role”; “contradictory messaging”; “Interactional dilemma”; “[irrelevance of] the usual diagnostic roadmaps”; diagnostic uncertainty “marshalled”	“A number of families in our study that were exceptions to [the] general rule [of how they understood the result and wished to be managed] [Perhaps due to] structural and cultural factors”
Timmermans and Buchbinder (2012) [25]	How similar to symptomatic infants? Treat as such?		Vigilance; preventative measures; “overreacting”	NBS programme not undermined	“Changed disease ontology”; “collectively negotiating the parameters of the anomaly”; “knowledge in the making”; “bridging work”	Parents’ internet use and inaccurate/ outdated information
DeLuca et al. (2011) [26]	“Living with a potential illness”; not a “normal life”; “something could happen”	“As if a death occurred”; “consumed by thoughts of the disorder”	Monitoring for symptoms	“Unshakable faith in the merits of newborn screening”	HCPs provide reassurance but some professionals also felt to have inadequate knowledge; importnace of personal approach	Impact of socioeconomic background; inadequate NBS knowledge; internet searching; persisting inaccuracies
Tluczek et al. (2010) [27]	Uncertainty and ambiguity; Abnormal result/ healthy child; “always going to be this cloud hanging over”	“Initial psychological plummet”; only “worrisome content” is heard; “Frightening emotional roller coaster”; “potential loss of infant”; “emotional suffering”; “always in the back of our mind”	“Action-oriented coping”; “mobilising”; “hyper-vigilance”; “precautionary health measures”	“A roller coaster ride of trying to figure out what it all meant”	“[Professionals] help reframe the situation”; “Structure Providers” [HCPs shape interpretations]	“The Opportunity–Danger Continuum”; gender differences; impact of “cognitive resources”; “Structure Providers” [social contacts/ media shape interpretations]; Need for “facts and figures”; Need new label that does not connote the ‘true’ disease
Hayeems et al. (2017) [28]	“Uncertainty about what an inconclusive diagnosis means”; Diagnosis v. “perfect” looking child; Fear of “potential labelling effects”	“Cascade of effects”	“Active symptom seeking”; monitoring; lifestyle changes	“Unsettled meaning”; struggle to make sense; “interested in how it all worked”; “value placed on knowledge unto itself, for most”;	“Mixed messages”; “Mixed feelings about surveillance”	
Sadat et al. (2020) [29]	“Uncertainty”	Anxiety; helplessness; fear of child’s death; concern about future	Continuation of unnecessary treatment	Appreciation of enhanced health awareness; stress caused by the NBS result “not justified”	Mixed feelings about ongoing monitoring/ treatment	
Johnson et al. (2019) [31]	“Liminality and Uncertainty”; “typical child” v. genetic designation; “don’t know what’s round the corner”; “there is that chance [of symptoms developing]”	Trauma; “Fear, Grief, and Threat to Child’s Life”; “bombshell”; extended issues; continued threat	“Prophylactic medical regimens”; “need to hyper-control the environment”; child’s health as “battle”; “all this prevention”	“Making certainty out of uncertainty”; “Utility of Labelling”; value of knowledge	“Challenging traditional ideas about health and illness”; “Incongruence with the Traditional Medical Model”; “moving the goalposts” [mixed messages]; “Intrusion & control”	“Information Sufficiency”; personal context; “Tension of Idiosyncratic Forms of Certainty”
Kerruish (2011) [32]	“Between planning for adversity and hoping for the best”; anticipation of potential medical complications/short life	“Generally mild” initially; “fluctuating recurrence of worries”; “subtle, complex, dynamic and ongoing reactions”	“Behavioural modification”; monitoring; “specific practical changes”; “preparing themselves for a disease that may never eventuate”	“Process of sense-making”; “conscious process of minimisation”; “comparison strategy” to make sense of risk; “Lucky to know”		“Varied” emotional responses between parent/ intra-individually/ over time; interaction of personal and contextual factors and uncertainty; "gist" of information
Tluczek et al. (2019) [30]	Envisioning a “normal” future; hope for “normal” life v. awareness of special health needs	Worry about future	“Protective behaviours”	Comparing child’s health to ‘a typical CF patient’		Complex interrelated factors influence parent perceptions of vulnerability; effect of depression and anxiety

### Data synthesis (Fig. 3)

**Fig. 3:**
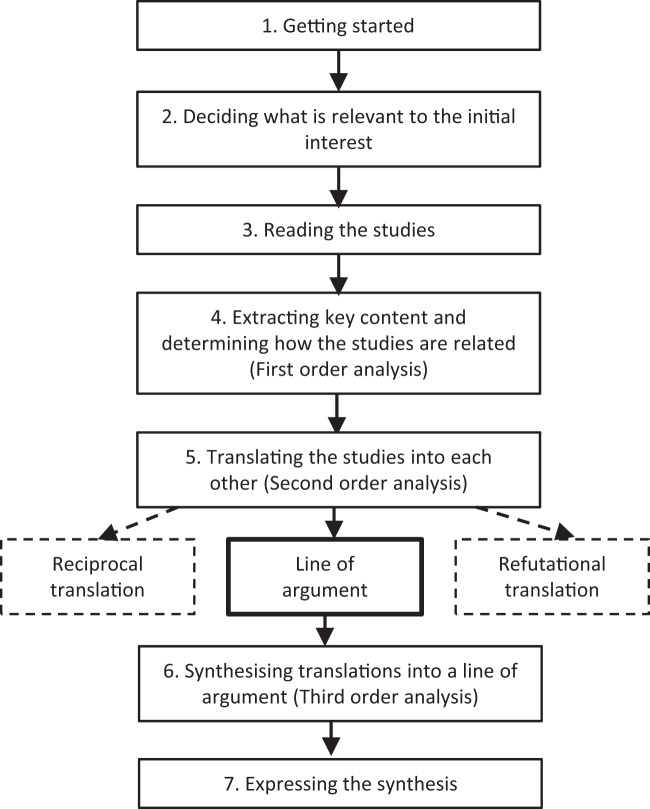
Meta-synthesis process. Flow diagram illustrating our use of Noblit and Hare’s [21] analytic strategy for meta-ethnography, using principles of first and second-order constructs [22].

This meta-synthesis employs principles of meta-ethnography [21] and first and second-order constructs [22] to amalgamate the essential phenomena of studies into a new, substantive interpretation [23]. Meta-ethnography is an interpretive method of research synthesis that compares how studies are related and what they say about the topic in each other’s terms [21]. The process is shown in Fig. 3. Each paper was read repeatedly, highlighting key ‘first order’ content (the study authors’ words) and grouping it according to conceptual similarity. At this stage an assumption is made about how studies are related: they may be directly comparable (reciprocal synthesis); oppose each other (refutational synthesis); or (the approach chosen here), taken together, enable a line of argument about the topic [21]. The key concepts were then unpacked and explored further – translating studies into one another [21] - in the second-order analysis to produce second-order constructs (‘constructs of constructs’) [22]. Third-order analysis distils these into a ‘line of argument’ [21] or theory (‘a whole among a set of parts’) [21].

## Results

### Search results

Searches identified 8815 articles (Fig. 2). Nine were eligible for inclusion.

### Study characteristics (Table 2)

Though we did not constrain our search to NBS, the aim of all studies was to explore the experience and impact of various uncertain NBS results (Table 2). Two [24, 25] specifically explored how uncertain results were navigated in the clinic. One [25] focused more on this than on emotional impact. All participants were parents of affected children (aged four months - eight years [parents of older children gave retrospective accounts]). Where demographics were given (five papers), most parents were married and in their thirties. In five studies [24–28], parents were interviewed/observed more than once. Of the mixed-methods studies[28–30], only qualitative data were synthesised.

### Quality appraisal results (Supplementary Table A)

Seven studies were high quality: consistently using appropriate methods of data collection and analysis, and describing research processes in sufficient detail. Eight demonstrated researcher reflexivity and insight. One [26] lacked clarity regarding the method of data analysis, however, this did not affect transferability of findings. One [29] was deemed to have ‘significant flaws’ [20] as it did not appear to use specific qualitative methods or give sufficient detail of the qualitative part of the study. This was not thought to affect the overall credibility of this meta-synthesis and so is still presented.

## Results of the synthesis

### First-order analysis: content issues and key concepts

Table 3 extracts the key content from the papers and groups according to conceptual similarity. Words in quotation marks are study authors’/participants’ original terminology, although some paraphrasing was necessary for brevity. These groups represent six key concepts: ‘Uncertainty and identity’; ‘Emotional response’; ‘Behavioural response’; ‘Cognitive response’; ‘Medicine and the role of professionals’; ‘Individual differences, communication and information needs’.

### Second-order analysis

Second-order interpretations (Column 2 of Table 4) were derived from the key concepts.

**Table 4:** Summary of meta-synthesis.

1. Key concepts		2. Second-order analyses		3. Third-order analysis
Uncertainty and identity *“you don’t know what’s round the corner for these kids, they’re doing fine now but in five, six years they might not be”* [31 (p. 8)]	➨	*Uncertain futures; liminal identities:* Prolonged uncertainty about the the clinical significance of the result and the impact (if any) it will have. Knowledge of result clashes with experience of apparently healthy child.	Features of the experience identified from the analysis suggest that…	
Emotional response *‘as if a death occurred*’ [26 (p. 56])	➨	*The emotional impact of a child’s uncertain screening result:* Strong negative emotional impact upon receipt of result and continuing long term in complex ways. Strong emotions may affect understanding of medical information.		
Behavioural response *“sleeping in her room with my hand on her back to make sure that I could feel her breathing”* [28 (p.169)]	➨	*Behavioural impact, and the impact of behaviour:* Coping with result by adopting behaviours such as ‘preventative’ measures, vigilance, lifestyle changes. Paradoxically may actually put focus on disease and perpetuate anxiety and ‘sick role’.		
				Uncertain screening results do not fit the traditional medical model
Cognitive response *‘a dynamic process of trying to make sense of the risk*’ [32 (p. 350)]	➨	*Cognitive appraisal of the meaning and value of the result:* Drive to understand and figure out what the result means for them, often by referring to other types of risk, and the perceived benefits of knowing about the result.		*(a model which implies one is either sick or healthy)*
				and therefore…
Medicine and the role of professionals ‘*collectively negotiating the parameters of the anomaly*’ [25 (p. 212)]	➨	*‘A new medical model’: bridging the gap*. New types of screening results cause uncertainty for laypeople and professionals alike. This may cause issues between patients and professionals, but may also present opportunities for new ways of working to collaborate and reframe uncertainty.		Uncertain screening results disrupt identity
Individual differences, communication and information needs “*The thing that stuck in my head was that sound and that horrible coughing”* [27 (p. 214)]	➨	*Individual and intra-individual differences in understanding and coping:* Between individuals (own experiences with disease; mental health; education; SES; language; culture); within individuals over time (contextual factors e.g. medical visits); within couples and families.		*(due to responses to result in context of traditional medical model)*

Author quotes are indicated in italics and participant quotes in quotation marks.

#### Uncertain futures, liminal identities

The screening results discussed contain a core ambiguity, which operates on multiple levels [24]: biologically as the clinical significance is unknown, and socially as it remains unclear what impact it will have on the child’s and family’s life. As little is known of the natural history or prognosis of these results [27] this ambiguity has *no conclusive endpoint* [24 (p. 416)]: “of all the people they’ve researched nothing has happened but something could happen. So it kind of leaves it wide open. I wonder if something is going to happen later on” [26 (p. 57)]. Parents were uncertain about their child’s long-term health: “you don’t know what’s round the corner for these kids, they’re doing fine now but in five, six years they might not be” [31 (p. 8]); “I think it is not just the insulin but the eyesight going and the potential for gangrene, and a shortened life, all those things” [32 (p. 350)]. This anticipation confers an identity which was labelled a *patient in waiting* [24]. This was apparent in many parents’ accounts of their children: “I want him to have a normal life. Right now he isn’t getting it” [26 (p. 58)]; “I was so excited for her to be healthy, and then to find out that there was always going to be this cloud hanging over her” [27 (p. 216)]. One study explored the identity of a ‘healthy child’ by asking parents to put their child on a scale of ‘completely healthy’ to ‘serious health condition’ [31]. Some placed them in between, whilst others said they were ‘completely healthy’ but “there is that chance [of symptoms developing]” [31 (p. 8)]. For many, the result was at odds with the child’s “perfect” appearance [28] - “if we didn’t know this, we would just assume he’s a healthy kid” [27 (p. 216)]—preventing a firm identity. Due to the result (“it’s one of those weird genes… he doesn’t have classic [cystic fibrosis (CF)] but we can’t say that he doesn’t have CF at all” [28 (p. 169)]), children are *not sick, but not normal* [24 (p. 416)] Parents worried about stigma—“we didn’t want her to be known as ‘that sick kid’” [27 (p. 218)]—and some children themselves were aware of differences: “we came home from the hospital last month and [18 month old] said, ‘am I sick?’” [28 (p. 169)]; “[4 year old] said she couldn’t tidy up because she’s got cystic fibrosis” [31 (p. 9)].

#### The emotional impact of a child’s uncertain screening result

The result had a significant emotional impact on the parents. Most felt this upon initial receipt of it: a “bombshell” [31 (p. 5)] causing an *initial psychological plummet* [27 (p. 214)] of fear, anxiety, and helplessness [29]. Many felt grief, *as if a death occurred* [26 (p. 56)] and worried their child would die [27, 29, 31], despite no such risk in these cases. This strong initial impact may affect understanding of information, so *only worrisome content* (such as the disease label) is heard [27 (p. 214)]: “I couldn’t even comprehend anything that [healthcare professional (HCP)] said. I couldn’t even function at the moment she was telling me” [26 (p. 56)]. Children across studies were up to eight years old, suggesting the impact of the result continues long-term, beyond the initial shock. This was described as an emotional roller coaster: [24, 27] a fluctuating recurrence of worries [32], causing a *cascade of effects* [28 (p. 168)] on families [28, 31]. There may be *subtle, complex, ongoing reactions* [32 (p. 351)] years later. Though uncertainty may permit optimism [32], the uncertainty remains unresolved, such that the result remains in the back of parents’ minds [26, 27, 31, 32].

#### Behavioural impact, and the impact of behaviour

Parents’ behavioural response to the result was characterised as *action-oriented coping* [27 (p. 217)], an adaptive shift or ‘mobilisation’ from the emotional stage: “Okay guys, suck it up” [27 (p. 217)]. They engaged in preventative behaviours, perhaps as *measures to offset the ‘real’ disease* [24 (p. 416)]. These included: “cleaning constantly” [31 (p. 6)]; prophylactic medical treatment: [29, 31] “I worry that something will happen if he stops taking it” [29 (p. 23)]; monitoring: “sleeping in her room with my hand on her back to make sure that I could feel her breathing” [28 (p. 169)]; and vigilance for potential symptoms, such as “that ketoney sort of smell associated with diabetes” [32 (p. 350)] Others included seeking reassurance from HCPs [24, 27, 31, 32], and restricting activities - “She has to be careful about where she plays” [31 (p. 7)]. This often extended to decisions about jobs and locale [24, 28]. Although *action-oriented coping* [27 (p. 217)] may seem positive, these disease-focused behaviours may actually *settle the condition as real disease in the lives of many parents of patients in waiting* [24 (p. 415)]. What began as strategies to manage uncertainty [27], or regain control, may perpetuate the distressing sense that the child is ill. This is described as a *dilemma between preparing themselves for a disease that may never eventuate, or choosing to ignore their child’s genetic risk, potentially missing the opportunity for such planning* [32 (p. 351)]: “all this prevention has stopped her getting ill, she could’ve been very poorly, we’ll never know will we” [31 (p. 9)].

#### Cognitive appraisal of the meaning and value of the result

Parents underwent a process of sense-making [32] about the result: “I think we were confused actually for quite a while… So it was just, it was like a roller coaster ride of trying to figure out what it all meant” [27 (p. 215)]. In the face of *unsettled meaning* [28], parents found ways of *making certainty out of uncertainty* [31]. This was an active process: [26, 27] “I was interested in how it all worked so I wanted to know all the details” [28 (p. 169)]. Parents evaluated the result by weighing it up against other scenarios (*a dynamic process of trying to make sense of the risk by locating it on a map of potential illnesses and situations they considered to be more serious or more likely* [32 (p. 350)]), including “full-blown” disease [28]. Many viewed the result as useful [26, 28, 31, 32] – “there would never be an occasion where I think, ‘oh, I wish I didn’t know’” [31 (p. 8)], or felt *lucky to know* [32 (p. 350)]. Their relationship to this was complex – “I’m very glad that we know about it, even though it kind of sucks” [28 (p. 169)]. Some did not appear to value it: “It doesn’t achieve anything […] There’s no effect on her life, no effect on our life” [31]. (p. 9) This may be deliberate minimisation: [32] “There’s plenty to worry about with your children so I am trying to minimise my worries as much as I can, not being overly neurotic and worried about things” [32 (p. 350)]. For some the harm of the result offset any benefit: “I feel that the amount of stress I experienced at the beginning was not justified since my child has never had any problems” [29 (p. 23)].

#### ‘A new medical model’: bridging the gap

The results appeared to be new territory for parents—*an ontological transformation of disease categories* [25] that is *incongruent with the traditional medical model* [31]. They expect certainty which is often not possible: “[HCP] wasn’t really sure what it was or anything” [26 (p. 56)]. This causes issues when parents get mixed messages by HCPs [24, 28, 31] “moving the goalposts” [31 (p. 9)]. Medical encounters may allow families and HCPs to *collectively negotiate* the uncertainty [25 (p. 212)]. This process (*bridging work* [25]) was apparent in parents’ accounts: “[HCP] was clearly prepared for our confusion, she handled it” [27 (p. 215)]; “it helped us understand” [26 (p. 57)]; “[HCP] was fantastic and explained the whole thing” [31(p. 7)] Where HCPs cannot resolve uncertainty completely, they may *reframe the situation* [27 (p. 214)]. Some parents had mixed feelings [28, 29], reporting ongoing monitoring was *emotionally draining* [28 (p. 168)]: “At some point this, looking for every single thing, has to stop” [28 (p. 169)]. Some stopped follow-up [24, 29], or were ‘at odds’ with HCPs over parents’ reluctance to drop precautions. Timmermans and Buchbinder describe an example of *bridging work* in this case:

The physician realised that asking a family to eliminate a preventive treatment that they had been using for years could be a difficult proposition. Instead, he opted to gradually phase out the treatment […], giving the parents time to adjust and to accept that their child was likely to be fine [25 (p. 217)].

A personal approach may help families negotiate uncertainty: “They treated us like humans, not just as a number, and talked to us in ways we would understand” [26 (p. 57)].

#### Individual and intra-individual differences in understanding and coping

While similarities were found across studies, unique responses within study populations suggest the same result can have varied effects [30]. Parents used a range of names ranging from the ‘true’ disease to a ‘mild’ or ‘borderline’ case. The results’ ambiguity meant parents arrived at various interpretations subject to individual factors. Many put it in context of their own personal experience with disease [27, 32]—“The thing that stuck in my head was that sound and that horrible coughing” [27 (p. 214)] and bereavement - “my dad died when I was 10, and I imagined [sibling] at that age at his brother’s funeral, and that was all that was going through my head at the time” [31 (p. 5)]. Mental health also appeared to influence how parents responded to the result [27, 31, 32]. Depression caused them to *filter out any messages of hope and to focus solely on the worst possible scenario* [27 (p. 216)]. There were different responses within couples [24, 27, 31]. This may damage the *emotional tenor of the relationship* [24 (p. 416)], and may perpetuate uncertainty about the result by forming disparate models as children grow. Within individuals, result perception may vary over time in response to contextual factors (such as clinic visits) [32] – disrupting any stability. Socioeconomic status may also affect coping: in one study, *parents who were younger, with less formal education, or were underserved minorities* [26 (p. 58)] had inaccurate understanding of results and more difficulty interacting with HCPs. Parents in this study *accessed primary research articles, and one parent contacted a nationally recognised researcher* [26 (p. 57)]: requiring education and social capital. While less educated parents initially struggled to understand the result, they may be less uncertain than more educated parents [27], suggesting the effect of education is complex. HCPs should be aware that language and culture may affect how results are perceived and understood [24].

### Developing a line of argument: third-order analysis

**Fig. 4:**
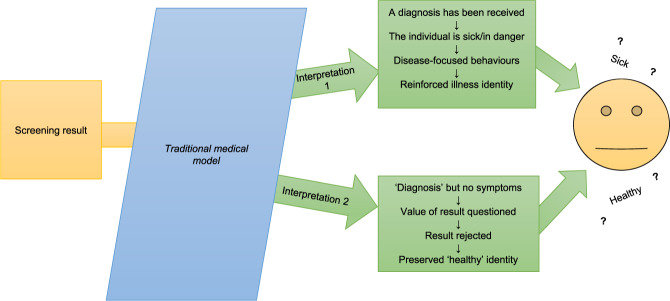
Third-order analysis (line of argument). This model illustrates the line of argument from the meta-synthesis: uncertain screening results, viewed through the lens of the traditional medical model, are interpreted in maladaptive ways that disrupt identity. The traditional medical model does not appear to facilitate perceptions of uncertain genetic screening results that are medically and psychologically beneficial and do not disrupt identity.

Interpretations from the meta-synthesis suggest potential explanations of the experience of receiving results of uncertain clinical relevance from screening (Fig. 4). Recipients may struggle to understand results using a traditional medical model, with its focus on ‘the diseased body' [33] and diagnosis of symptoms [34]. For example, parents referred to the results as a ‘mild’ or ‘borderline’ case of disease, and contextualised them in terms of their experiences with ‘traditional’ illness. Although academics and HCPs refer to the uncertainty of the result, within the traditional medical model there is no perceived uncertainty for most—just a binary between ‘ill’ and ‘healthy’ [10]. This may explain the intense impact: parents receiving abnormal screening results make sense of them using a model that implies a diagnosis; and receiving a diagnosis is traumatic. What follows also belongs to a traditional medical model (e.g. hospital visits), reaffirming the disease as a concrete entity. Interpreted via this model, the result triggers parents to live as if the child has a disease, for example making substantial lifestyle changes is akin to how a child’s illness may shape family life. They appear comforted by enacting traditionally ‘medical’ behaviours, such as giving treatments or restricting activities, and fear serious illness or death if these are not adhered to. Due to the nature of the result, however, there can be little reassurance that these ‘preventions’ work, perpetuating a sense of an unwinnable battle. Practicing illness-focused behaviours within the traditional model constructs an ‘illness identity’—the child has a ‘diagnosis’, sees specialist HCPs, and may take medicine or have activities restricted: ergo they are ‘sick’. Yet their identity is upset by the uncertain nature of the result, putting them somewhere between ‘sick’ and ‘healthy’. This upset may contribute to the emotional impact of the screening result; persisting because the disrupted identity remains unresolved. Confusion and distress results from being in the medical world without symptoms. Those who disavow the screening result do so because their child has no symptoms: the result has no value as it does not have the explanatory power of a traditional diagnosis. Negating the result affirms a ‘healthy child’ identity, though this is unstable. The traditional medical model does not appear to allow awareness of uncertain genetic screening results in a way that is medically and psychologically beneficial and does not risk disrupting identity.

## Discussion

This review exposes concerns about the negative consequences of screening results of uncertain clinical relevance, which overlap with bioethics literature [8, 9] and may be shared by HCPs [35]. Responses involved illness-focused practices that raised anxiety and reinforced a sick role: against advice that management must not medicalise and “draw [the individual] into an illness model” [9 (p. 5)]. Though medical uncertainty is not new [36], advances in screening may increase results of uncertain clinical relevance, on a wider scale [4]. It is therefore important to ensure that people can cope in healthy ways.

Interpretations from the meta-synthesis suggest that these parents struggled to understand the results using their current medical model. In this model, observable symptoms are the basis for diagnosis: [34] a cognitive schema that organises and directs our encounters with medicine [24]. For uncertain result recipients, this ‘diagnosis’ schema is missing, affecting ability to navigate the medical world. Hence, uncertain screening results signify a “syntactical reorganisation of disease in which the limits of the visible and the invisible follow a new pattern” [34 (p. 195)]. Parents felt tension between their child’s ‘healthy’ identity and the genetic result—compounded by reminders (e.g. clinic visits) of an ‘illness’ identity. That these results could affect identity thus reflects the idea of ‘patients in waiting’: [24] a new population emerging from screening. Here, the result became an “interpretive frame” [37] which threatened the identity of the child and family. Negative affective response to uncertain results is found elsewhere in the literature [10, 38]. Emotional state affects response to uncertainty in uncertainty management [39] and uncertainty in illness theories [40]. HCPs should ensure result communication is clear to minimise distress. Still, distress could be prevented outright if people had a prior model with which to assimilate information about uncertainty and genomics in a healthy way. Although care may be taken to ensure that the names applied to uncertain results are helpful descriptive terms rather than diagnostic labels [41], these may still be seen as de facto diagnoses without the schemas to integrate them. The idea that uncertain genetic results are subjectively interpreted through a ‘lens’ of individuals’ prior expectations, beliefs, and experiences is found elsewhere in the literature [10, 38]. While uncertainty is not unusual for HCPs [10, 35], data suggests patients do not expect uncertain results; [10] also that tolerance of uncertainty has reduced as medicine has been seen to progress [36]. Explicit recognition of uncertainty may reduce heterogeneous subjective responses [38] and improve resilience [42]. In predictive genetic testing, understanding of and adaptation to uncertainty is achieved with pre-test counselling which includes in-depth discussions about the possible outcomes of testing and its meaning [42, 43]. However, as discussed, there are differences between testing and screening: for example, NBS may be passively undergone due to feelings of routinisation [13] or an ‘implicit contract’ [44], with differences how pre-screening information is accessed and attended to [14]. Furthermore, as genomic screening becomes far more widespread, the time and resources required for in-depth counselling of every individual may be challenging [43]. Genetic counselling is “to help people understand and adapt to the medical, psychological, and familial implications of genetic contributions to disease” [45 (p. 77)]. One idea could be to scale up some of the goals and techniques of genetic counselling into a public health approach to enhance genetic literacy in the general population [46]. There are initiatives to develop genomic fluency for HCPs by NHS England [47]. Initiatives should be developed with approaches like patient and public involvement (PPI) to ensure that messages are acceptable and effective [48].

### Strengths and limitations

We have provided insight into parents’ experiences of receiving uncertain results for their children from NBS. NBS entails unique concerns due to impact on parent-child relationships [49]. Our search, while not solely for NBS, suggests there are currently no studies of other programmes that meet the criteria of this review. Adult screening programmes are arguably targeted at sub-populations who are aware of increased risk [3], whereas NBS is offered universally [13, 14]. Nonetheless, there will be experiences not covered. Receiving a result for a child is different to for oneself. Research on adults’ experiences of receiving unexpected uncertain screening results for themselves is required; opportunities are likely as new programmes and technologies emerge. Within genetics there are different types of uncertain results, requiring a nuanced response as appropriate. Looking at the qualitative research has allowed deep exploration, which can give a richer understanding of impact. Still, a wealth of relevant quantitative research was not included in this review. Of the nine papers, just one was from the UK. This suggests our findings may represent a universal response to this issue, regardless of country and healthcare system. Nonetheless, as genetics is to be further integrated into the UK National Health Service [4], further UK-specific research may identify pertinent issues. We suggest the need for a new model to contextualise uncertain screening results. However, there may be barriers to this, such as the challenges of risk communication. Also, while we suggest receiving a ‘diagnosis’ is traumatic, labels can help psychosocially and in accessing support [50]. However, our results echo the idea that diagnostic labels, once invoked, cannot easily be revoked [50]. These complex issues require further research. Qualitative methods are apt to explore this evolving landscape.

## Conclusions

This review suggests that receiving a screening result of uncertain clinical relevance from NBS can be distressing, and that negative impact may persist due to unresolved uncertainty. The meta-synthesis suggests individuals are driven to resolve uncertainty, however, responses vary and may cause further harm. We suggest that, currently, uncertain results may cause distress because recipients interpret them using models which imply the result is essentially a diagnosis and therefore one is ill. In practice, there must be clear initial communication that there are no immediate health implications from this type of result. Where further investigations may be needed, this should be introduced without unnecessarily invoking medicalisation or threat. Future research should focus on public understanding of a new medical model that accounts for genomics in a way that maximises benefit and reduces potential psychological harm.

## Supplementary information

Supplementary Table A

## Data Availability

Data sharing not applicable: no datasets were generated/analysed in the current study.
